# Functional Analysis of the Phosphate Transporter Gene *MtPT6* From *Medicago truncatula*

**DOI:** 10.3389/fpls.2020.620377

**Published:** 2021-02-04

**Authors:** Yuman Cao, Jinlong Liu, Yuanying Li, Jing Zhang, Shuxia Li, Yunru An, Tianming Hu, Peizhi Yang

**Affiliations:** College of Grassland Agriculture, Northwest A&F University, Yangling, China

**Keywords:** *MtPT6*, *Medicago truncatula*, phosphate transporter, low phosphate stress, nodule

## Abstract

Phosphorus is one of the essential macronutrients required by plant growth and development, but phosphate resources are finite and diminishing rapidly because of the huge need in global agriculture. In this study, 11 genes were found in the *Phosphate Transporter 1* (*PHT1*) family of *Medicago truncatula*. Seven genes of the *PHT1* family were available by qRT-PCR. Most of them were expressed in roots, and almost all genes were induced by low-phosphate stress in the nodule. The expression of *MtPT6* was relatively high in nodules and induced by low-phosphate stress. The fusion expression of *MtPT6* promoter-*GUS* gene in *M. truncatula* suggested that the expression of *MtPT6* was induced in roots and nodules by phosphate starvation. In roots, *MtPT6* was mainly expressed in vascular tissue and tips, and it was also expressed in cortex under low-phosphate stress; in nodules, it was mainly expressed in vascular bundles, cortical cells, and fixation zone cells. MtPT6 had a close relationship with other PHT1 family members according to amino acid alignment and phylogenetic analysis. Subcellular localization analysis in tobacco revealed that MtPT6 protein was localized to the plasma membrane. The heterologous expression of *MtPT6* in *Arabidopsis* knockout mutants of *pht1.1* and *pht1.4* made seedlings more susceptible to arsenate treatment, and the phosphate concentrations in *pht1.1* were higher in high phosphate condition by expressing *MtPT6*. We conclude that *MtPT6* is a typical phosphate transporter gene and can promote phosphate acquisition efficiency of plants.

## Introduction

Phosphorus is one of the most important macronutrients for plant growth and development because it is the key constitution of cellular components and involved in many metabolic processes (Schachtman et al., 1998; Horst, 2012). Inorganic phosphate (Pi) is the only form that plant roots can assimilate. However, Pi in soil is commonly suboptimal for plant growth because of the interaction with cations and the conversion into organic forms by microorganisms (Hinsinger, 2001; Vance, 2010; Lizbeth et al., 2014). To ensure crop productivity, the excessive application of fertilizers has become common in agriculture, leading to many environmental problems (Domagalski et al., 2007). Thus, breeding plant varieties with high Pi acquisition and utilization is urgently needed to reduce fertilizer and enhance agricultural sustainability.

Plants have developed multiple transporters, including PHT1, PHT2, PHT3, and PHT4 family, involved in Pi uptake by root and remobilization in plants to adapt to low phosphate conditions (Liu et al., 2011). Phosphate uptake from soil by root cells is the first and essential step in the Pi assimilation process, and the PHT1 family is responsible for the uptake. The concentration of Pi in plant cells is much higher than that in soil; thus, plants have to uptake Pi against a sharp concentration gradient. PHT1 family members have been identified in many plants. Nine and 13 genes have been identified in *Arabidopsis* (*AtPHT1;1–9*) and rice (*OsPT1–13*), respectively (Karthikeyan et al., 2002; Paszkowski et al., 2002; Liu et al., 2011). In *Arabidopsis*, the expression levels of *AtPHT1;1* and *AtPHT1;4* are highest in root tissues when seedlings are treated with low-phosphate stress (Karthikeyan et al., 2002). When these two genes are knocked out, the plant’s ability of Pi uptake is decreased significantly (Shin et al., 2004). In rice, *OsPT1* and *OsPT8* are constitutively expressed in shoots and roots, playing roles in Pi acquisition and maintenance of Pi homeostasis (Jia et al., 2011; Sun et al., 2012). *OsPT2* is a low-affinity transporter gene and associated with Pi translocation from roots to shoots (Ai et al., 2009). *OsPT6*, *OsPT9*, and *OsPT10* are highly induced by Pi starvation and play roles in Pi acquisition and translocation (Ai et al., 2009; Wang et al., 2014). Also, PHT1 family genes have been reported in wheat (Teng et al., 2017), barley (Preuss et al., 2010), maize (Nagy et al., 2006), and *Brassica napus* (Ren et al., 2014). Most members of the PHT1 family are mainly expressed in root tissues, and some are also expressed in shoots, which are responsible for the uptake and translocation of Pi (Mudge et al., 2002; Wang et al., 2014).

In *Medicago truncatula*, a model legume, five members have been identified in the PHT1 family. The *MtPT1* and *MtPT2* showed root-specific expression, while *MtPT3* and *MtPT5* were expressed in roots and aerial tissues. The expressions of all these four genes were induced by phosphate starvation (Chiou et al., 2001). *MtPT4* was vital for the symbiosis between mycorrhizal fungi and *M. truncatula* and specially expressed in arbuscular roots that contributes to the uptake of Pi (Harrison et al., 2002; Javot et al., 2007). The symbiosis between rhizobium and legume is also very important for plant growth and sustainable development of the environment. There are some studies showing that PHT1 family members of soybean play roles in rhizobium–soybean symbiosis. For example, *GmPT5* and *GmPT7* were found to promote nodulation (Qin et al., 2012; Chen et al., 2019). *GmPT7* also could improve Pi content in nodules (Lu et al., 2020). However, *M. truncatula* as an important model plant for legume, little is known about the function of PHT1 family genes in symbiosis with rhizobium. In our study, we found that there were at least 11 members in the PHT1 family of *M. truncatula*, and the expression levels of some genes were induced by phosphate deficiency. Moreover, *MtPT6*, an unreported gene, contributed to Pi uptake and translocation and might also be related to homeostasis in nodules.

## Materials and Methods

### Growth of *Medicago truncatula*

The seeds of *M. truncatula* (R108) were sterilized with 70% ethanol for 5–7 min and washed four times with sterilized water. Seeds with scarified seed coats were put in the fridge at 4°C for 2 days and germinated at 23°C. Then, seedlings were grown with hydroponic culture in growth chambers under 16-h light (25°C)/8-h (22°C) dark conditions. For hydroponic culture, we followed the system described by Tocquin et al. (2003) with NaH_2_PO_4_ as the Pi source (Liu et al., 2015). Nutritional solution was changed every 3 days. After 1 week, the seedlings were inoculated with rhizobium (*Sinorhizobium meliloti*, code No. 17726 and provided by Agricultural Culture Collection of China). The 5-week-old seedlings were treated with a low (5 μM; LP) or normal (260 μM; NP) phosphate concentration for 1 week. Shoots, roots, and nodules were collected and immediately frozen in liquid nitrogen and stored at −80°C.

### Extraction of Total RNA and qRT-PCR Analysis in *Medicago truncatula*

The total RNA of shoots, roots, and nodules from *M. truncatula* was extracted using MiniBEST Plant RNA Extraction Kit (Takara, Japan). Reverse transcript reaction was completed using HiScript II Q RT SuperMix for qPCR (+gDNA wiper) (Vazyme Biotech, China). qRT-PCR was performed using PrimeScriptTM RT Master Mix Kit (Takara, Japan) and Roche LightCycler 480. *Actin-7* was used as a control. Primers of all genes was shown as follows. *MtActin-7*, 5′-TTGCTGACCGTATGAGCAAG-3′ and 5′-TGGATGGACCAGACTCATCA-3′; *MtPT1*, 5′-TGGCAAAT CATAGTCGCATC-3′ and 5′-TGACATTTGTGAGGCTGAGG-3′; *MtPT2*, 5′-TGTTAGGTAAAGCCCGTGTTACT-3′ and 5′-AC GATGTTCCACACTTGGCT-3′; *MtPT5*, 5′-AAAGTTGGTGA TGGCAGGAC-3′ and 5′-TACACCGGGAGGAAGAACAC-3′; *MtPT6*, 5′-CAGGATAGGCGTGCAAGACA-3′ and 5′-TAAG CGTTGTCGCTCTAGCA-3′; *MtPT7*, 5′-AGTACCCGAGGCAA ATGGAA-3′ and 5′-GCTTAAGTGCTCCCCTACCA-3′; *MtP T8*, 5′-ACTGCTTCTCTTGGGTGTGG-3′ and 5′-AAAGACG TGACAGGGGAAGG-3′; *MtPT9*, 5′-GCCTGAGGCAAAGGG AAGAT-3′ and 5′-CTTCGTTGAAAGCACTCCCC-3′.

### Construction of Phylogenetic Tree

The amino acid sequences of MtPT1, MtPT9, AtPHT1, AtPHT4, GmPT5, and GmPT7 were aligned with clustalW method by Mega X 10.1 version. The phylogenetic tree was constructed by using the Neighbor-Joining method with bootstrap value 1,000 (Jones et al., 1992; Stecher et al., 2020).

### Vector Construction and Gene Transformation

For the constructing vectors to complement *Arabidopsis* knockout mutants, the 2×35S promoter cassette and green fluorescence protein (GFP) were cloned into pCAMBIA1300 vector, named 2×35S-pCAMBIA1300. Then, the coding sequence of *MtPT6* was cloned into 2×35S-pCAMBIA1300 vector between promoter and GFP, named *MtPT6*-pCAMBIA1300. These two vectors were transformed into *Agrobacterium tumefaciens* strain GV3101 by electroporation.

To construct the *MtPT6* promoter-*UidA* vector, 2,604-bp region upstream of the translation initiator, ATG, of *MtPT6* was cloned into pBI101 plasmid. The vector was transformed into *M*. *truncatula* roots by *Agrobacterium rhizobium* (Chabaud et al., 2006).

### Transgenic *Arabidopsis* Generation and Phenotype Identification Treated With Arsenate and Low Pi Concentration

*Arabidopsis* knockout mutants, *pht1.1* and *pht1.4*, were complemented with *MtPT6* gene by floral dipping (Davis et al., 2009). Transgenic plants were then selected *via* phosphinotricin resistance.

Knockout mutants and completed plants were germinated on 1/2 MS culture for 10 days. For arsenate (V; As) treatment, seedlings of *pht1.1* and complemented *pht1.1* with *MtPT6* genes were transformed to 1/2 MS culture with 400 μM As, while seedlings of *pht1.4* and complemented *pht1.4* were transformed to 1/2 MS culture with 300 μM As. For low phosphate treatment, seedlings were transformed to 1/2 MS culture with 5 μM Pi to identify their phenotypes.

### Subcellular Localization of Genes and Histochemical Localization of β-Glucuronidase Expression

*MtPT6* were co-expressed with the pm-rk, a plasma membrane marker, in *Nicotiana benthamiana* by *A. tumefaciens* infiltration. The fluorescence was detected with confocal microscope (LECIA TCS SP8, Germany). For protoplast isolation, about 1 g agro-infiltrated tobacco leaves was cut into 1-mm-wide strips and treated with 20 ml of MCP (500 mM sorbitol, 1 mM CaCl_2_, and 10 mM MES-KOH, pH 5.3) supplemented with 0.8% (w/v) Cellulase R10, 0.1% (w/v) Pectolyase Y23, and 0.5% bovine serum albumin (BSA) at room temperature for 2 h. Protoplasts were filtered through a 75-mm nylon mesh, concentrated at 100 g for 5 min, washed twice with MCP, and resuspended in MCP.

Histochemical β-glucuronidase (GUS) assays were performed following the protocol of β-Galactosidase Reporter Gene Staining Kit (Solarbio, China). When transgenic roots were 3-cm long in Fahraeus agar culture, seedlings were transformed to hydroponic culture with a normal phosphate concentration (260 μM). After 1 week, the seedlings were inoculated with rhizobium (*Sinorhizobium meliloti*). Seedlings were treated with a low (5 μM; LP) or normal (260 μM; NP) phosphate concentration for 1 week after 2-week inoculation with rhizobium. Roots were collected to do histochemical GUS staining.

### Measurement of Phosphate Concentration

Ten-day-old seedlings on 1/2 MS culture were then cultured hydroponically with a normal (130 μM; NP) phosphate concentration for 2 weeks. Plants were grown on hydroponic culture with different phosphate concentrations (5 μM, LP; 130 μM, NP; 1 mM, HP). Na^+^ concentration was balanced with NaCl because of the different concentrations of NaH_2_PO_4_. After 1 week, the shoots were collected to determine phosphate concentration with the ascorbate-molybdate-antimony method (John, 1970).

### Statistical Analysis

The experiments were repeated three times. The statistics were analyzed with SPSS Statistical 20.0 software, and differences between different groups were calculated with Duncan’s test. Significant differences were represented by ^∗^ for *P* ≤ 0.05.

## Results

### Phylogenetic Analysis of the PHT1 Family

**FIGURE 1:**
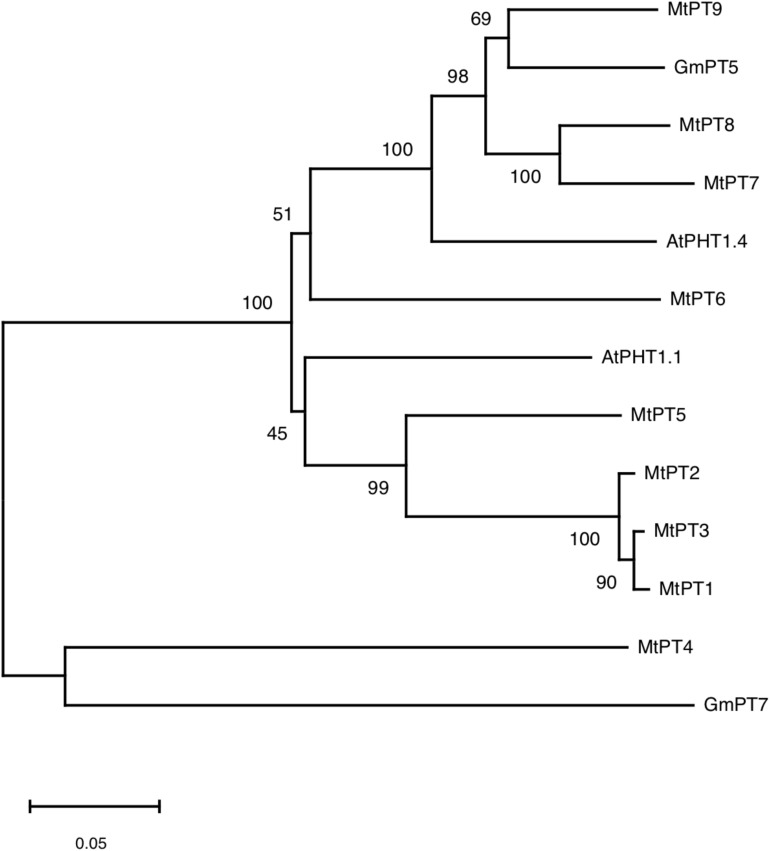
Phylogram of phosphate transporters of *Medicago truncatula*, *Arabidopsis*, and soybean.

According to the result of BLAST on NCBI^1^, 10 genes were found to be similar to *MtPT5* with the lowest similarity of nucleotide acid of 66% in *M. truncatula*, but only five members (*MtPT1*–*MtPT5*) have been reported. Amino acid sequence alignment indicated that the deduced amino acid sequence of PHT1 family genes had a high similarity to each other in *M. truncatula*, *Arabidopsis*, and soybean (Supplementary Figure 1). Phylogenetic analysis of amino acids showed that the relationship of MtPT1, MtPT2, and MtPT3 was closest, and MtPT5 was also closer to them (Figure 1). Moreover, AtPHT1.1 was close to these four proteins. The relationship of MtPT7 and MtPT8 was close; GmPT5 and MtPT9 were also close. AtPHT1.4 was relatively close to these four proteins, and MtPT6 belonged to the same branch with them. GmPT7 was close to MtPT4, which was related to mycorrhizal symbiosis.

### Expression Patterns for the PHT1 Family of *M. truncatula* Response to Low Phosphate Conditions

**FIGURE 2:**
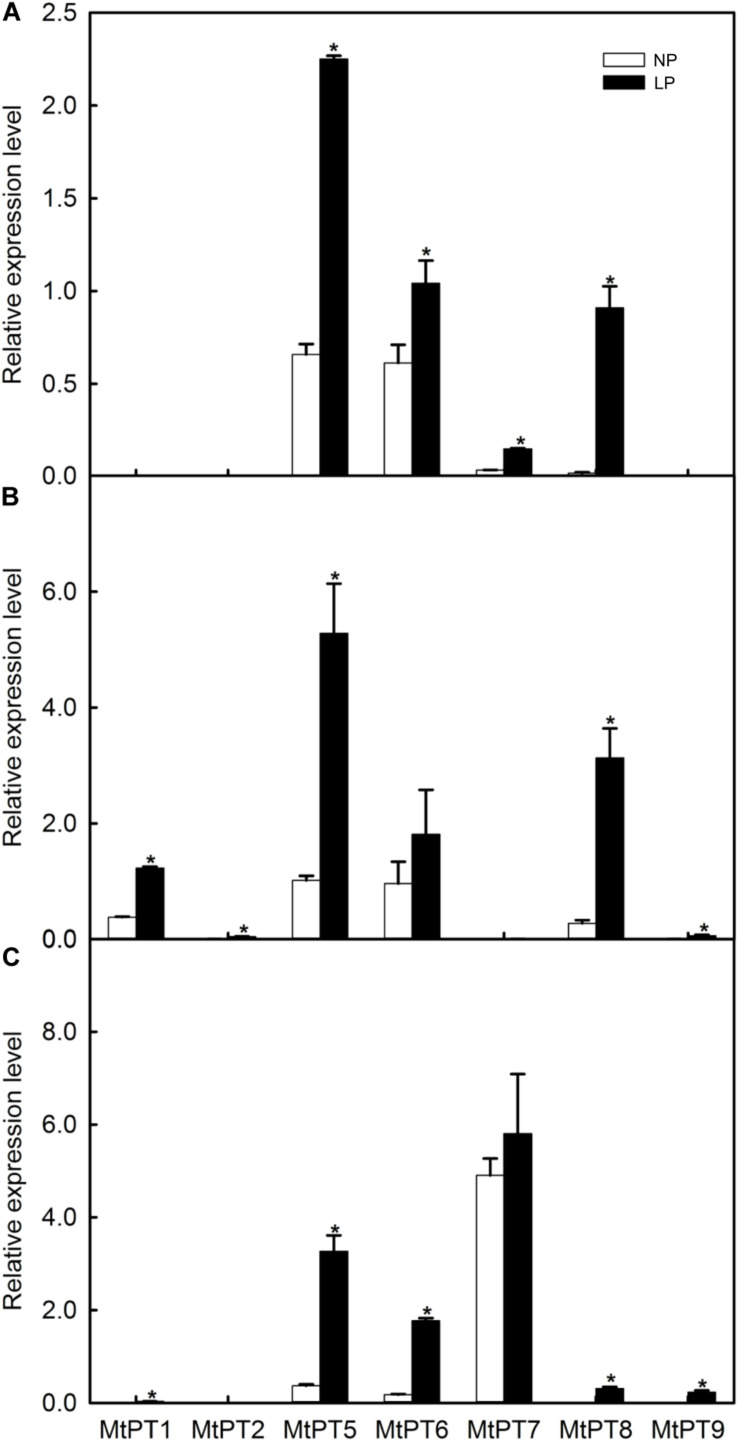
Effect of low phosphate treatments on the expression levels of PHT1 family genes in different tissues of *Medicago truncatula*. **(A)** Shoot; **(B)** Root; **(C)** Nodule. Relative expression of all genes in different tissues was normalized by the expression level of *MtPT5* in roots. NP, normal inorganic phosphate (Pi) concentration (260 μM); LP, low Pi concentration treatment (5 μM). Bars represent the mean ± SE (*n* = 3). Asterisks represent significant differences between NP and LP: *P < 0.05.

To analyze the expression response to low phosphate conditions, qRT-PCR was performed to detect the expression patterns of the PHT1 family in shoots, roots, and nodules. As shown in Figure 2, seven members of the *PHT1* family can be detected. Among these seven genes, three have been reported including *MtPT1* (MTR_1g043220), *MtPT2* (MTR_1g043290), and *MtPT5* (MTR_1g074930), and the remaining four genes are not reported including *MtPT6* (MTR_3g082700), *MtPT7* (MTR_7g096870), *MtPT8* (MTR_7g096880), and *MtPT9* (MTR_1g069935). Four members of the PHT1 family cannot be detected including two reported genes, *MtPT3* (MTR_1g043200) and *MtPT4* (MTR_1g028600), and two non-reported genes, MTR_1g069930 and MTR_1g074940.

The expression levels of most *PHT1* family genes were improved after LP treatment (Figure 2). *MtPT1* was mainly expressed in roots in the NP condition, and the expression level was significantly improved (3.44-fold) in roots after LP treatment. The results are similar to previous research (Liu et al., 1998, 2008). *MtPT1* was also detected in nodule tissues in the LP condition, although the expression level was low. It was reported that the expression level of *MtPT2* gene in roots was decreased with the increase of Pi concentration by Northern blotting; it was not expressed after seedlings were treated with 1 or 5 mM of Pi (Liu et al., 1998). Similarly, our results showed that its transcript level cannot be detected in NP conditions, but it increased significantly in roots after LP treatment. *MtPT5* was expressed in shoots, roots, and nodules, and its expression levels were increased in all tissues after LP treatment (3. 42-, 5. 29-, and 8.85-fold in shoots, roots, and nodules, respectively). Liu et al. (2008) also reported that *MtPT5* was expressed highly in roots, and its expression was decreased immediately after adding Pi to hydroponic medium. In the NP condition, *MtPT6* was expressed in all tissues, and it was relatively high in roots but low in nodules. However, its expression level was increased about 10-fold in nodules after LP treatment. *MtPT7* was mainly expressed in nodules, but the expression level was not increased significantly after LP treatment; it was not expressed in roots but in shoots, although it was relatively low. *MtPT8* was expressed in all tissues, and its expression level was relatively high in roots but low in shoots and nodules. After LP treatment, its expression levels were increased in all tissues. However, the expression level of *MtPT8* in nodules was lower compared with *MtPT5*, *MtPT6*, and *MtPT7*. *MtPT9* could not be detected in the NP condition. After LP treatment, it was expressed in roots and nodules, although the expression levels were relatively lower. In conclusion, the expression levels of *MtPT5* and *MtPT6* in roots and nodules were higher and induced by the LP treatment. *MtPT5* has been reported to be a high-affinity phosphate transporter gene of the *PHT1* family. However, the function of *MtPT6* has not been reported.

### Tissue Localization of *MtPT6*

**FIGURE 3:**
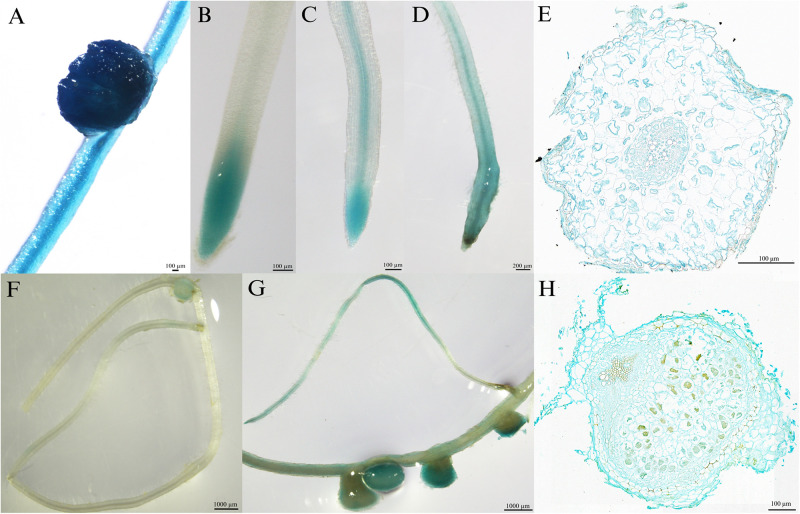
Tissue-specific expression of *MtPT6* in the roots and nodules of *Medicago truncatula*. **(A)** Expression of 35S promoter::β-glucuronidase (GUS) in roots and nodules in the condition of normal inorganic phosphate (Pi) concentration (260 μM). **(B)** Expression of *proMtPT6*::GUS in roots after hydroponic culture for 1 week. **(C)** Expression of *proMtPT6*::GUS in roots after hydroponic culture for 3 weeks. **(D)** Expression of *proMtPT6*::GUS in roots after low Pi treatment (5 μM) for 1 week. **(E)** Cross section of root after low Pi treatment for 1 week. **(F,G)** Expression of *proMtPT6*::GUS in nodules in the condition of normal Pi concentration (260 μM; **F**) and low Pi concentration (5 μM; **G**). **(H)** Cross section of nodule after low Pi treatment. Bars = 200 μm for **(D)**, 1,000 μm for **(F)** and **(G)**, and 100 μm for all other images.

To investigate the tissue localization of *MtPT6* further, the 2,604-bp region upstream of ATG was cloned to drive GUS reporter gene. The histochemical GUS staining results showed that the GUS reporter gene derived by 35S promoter could be expressed everywhere in the roots of *M. truncatula* (Figure 3A). *MtPT6* was mainly expressed in root tips and expressed lower in vascular tissues when seedlings were cultured hydroponically with normal phosphate concentration (260 μM) for 1 week (Figure 3B). After hydroponic culture for 3 weeks, *MtPT6* expression was increased in root tips and vascular tissues (Figure 3C). *MtPT6* was highly induced in low phosphate conditions (5 μM); it was expressed not only in root tips and vascular tissues but also in cortex (Figures 3D,E). This is similar to the results of qRT-PCR of *MtPT6*. These results showed that *MtPT6* might mainly play a role in phosphate uptake in young root tips, while it might also play roles in phosphate translocation with the growth of plants or in low-phosphate stress. As shown in Figures 3F,G, the expression of *MtPT6* was low in nodules, but it was strongly induced after low phosphate treatments. *MtPT6* expression was mainly observed in vascular bundles, cortical cells, and fixation zone cells (Figure 3H). These revealed that *MtPT6* is likely to be involved in phosphate homeostasis maintenance in nodules under low phosphate conditions.

### Subcellular Localization of MtPT6 Protein

**FIGURE 4:**
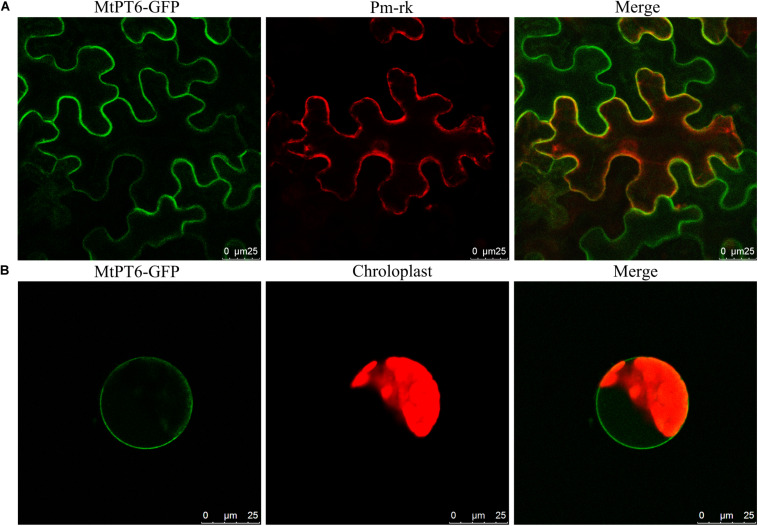
Subcellular localization of *MtPT6* gene. **(A)** MtPT6-GFP signal overlaid with a protoplast marker (pm-rk) in epidermal cells of tobacco leaves. **(B)** A protoplast isolated from mesophyll of tobacco. Scale bar, 25 μm.

For subcellular localization analysis of MtPT6, the MtPT6-GFP and a plasma membrane marker were co-expressed in *N. benthamiana* leaves. The results showed that MtPT6 could overlap perfectly with the plasma membrane marker not only in mesophyll cells (Figure 4A) but also in protoplasts (Figure 4B). So, *MtPT6* protein was localized to the plasma membrane.

### Heterologous Expression of *MtPT6*-Enhanced Sensitiveness to Arsenate in *Arabidopsis*

**FIGURE 5:**
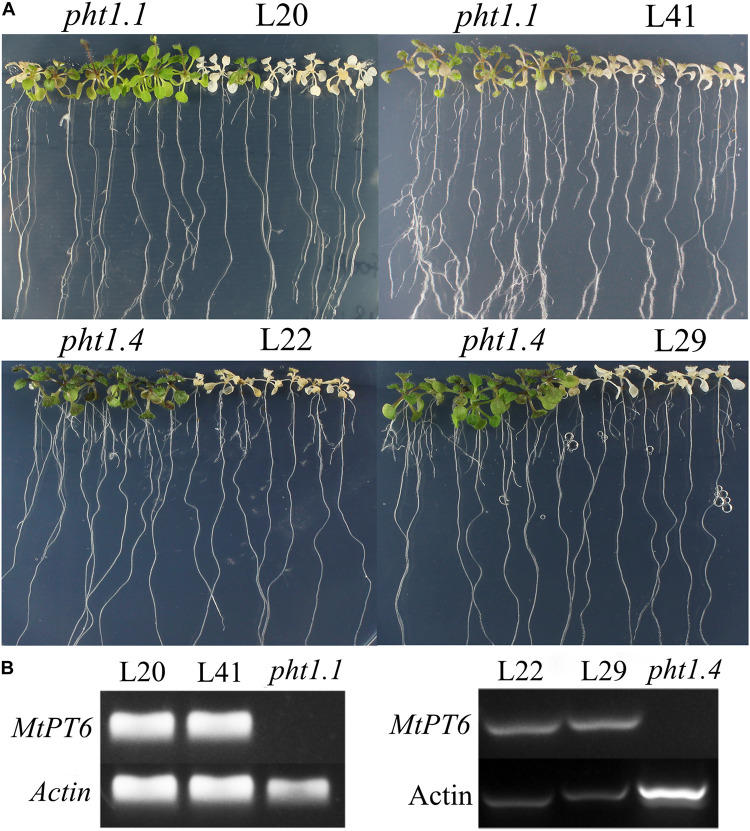
Phenotype of tolerance to arsenate treatment in different plants. **(A)** Phenotype identification of transgenic plants. **(B)** Identification of transgenic plants by PCR. *Pht1.1* and *pht1.4* are *Arabidopsis* knockout mutants; L20 and L41 are complemented *pht1.1* with *MtPT6*; L22 and L29 are complemented *pht1.4* with *MtPT6*. Pht1.1 and pht1.4 transgenic lines were treated with 400 and 300 μM As, respectively.

Arsenate can be taken up by phosphate transporters because of the structural similarity between arsenate and phosphate (Fitz and Wenzel, 2002; Shin et al., 2004). So, the ability of phosphate absorbance can be visualized by arsenate treatment in plants. Although *Arabidopsis* phosphate transporter knockout mutant *pht1.1* and *pht1.4* showed normal morphology and development in normal conditions with wild-type plants, they were more tolerant to arsenate (Shin et al., 2004). To test the effect of *MtPT6* on phosphate absorbance, *MtPT6* was ectopically expressed in *Arabidopsis* mutant *pht1.1* and *pht1.4*. We found that complementary plants by *MtPT6* were more sensitive to arsenate treatment than *pht1.1* and *pht1.4* seedlings (Figure 5A). To further confirm that the sensitiveness to arsenate was caused by *MtPT6* expression, DNA of transgenic lines was extracted to do PCR. The results showed that *MtPT6* was really expressed in these sensitive lines (Figure 5B).

### Heterologous Expression of *MtPT6* Enhanced Pi Uptake in *Arabidopsis*

**FIGURE 6:**
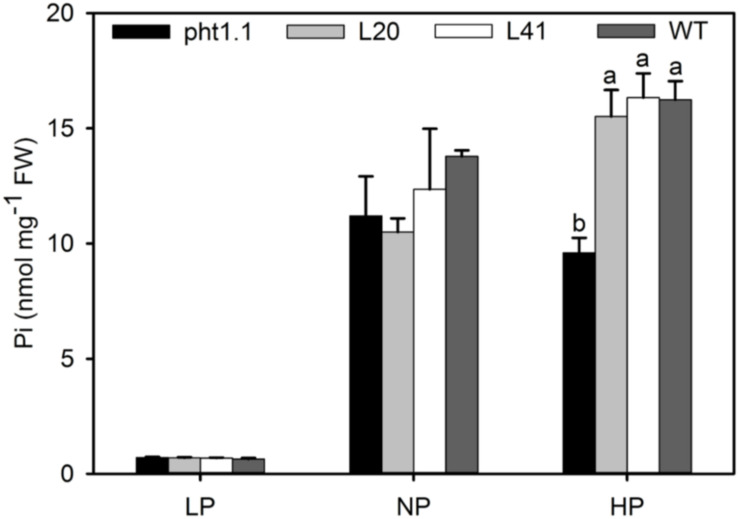
Phosphate concentrations of shoots of different lines under various phosphate levels. Effect of *MtPT6* on phosphate concentration of *Arabidopsis* knockout mutant *pht1.1*. LP, low inorganic phosphate (Pi) concentration treatment (5 μM); NP, normal Pi concentration treatment (130 μM); HP, high Pi concentration treatment (1 mM). Bars represent the mean ± SE (*n* = 3). Different letters represent significant differences according to Duncan’s test (*P* < 0.05).

To further investigate the function of *MtPT6* in Pi absorbance, the 2-week-old seedlings were treated with 5 μM (LP), 130 μM (NP), and 1 mM (HP) Pi of hydroponic culture for 1 week. Then, shoots were collected to measure Pi content. As shown by Figure 6, there was no significant difference between wild type, *pht1.1*, and complemented *pht1.1* under the LP treatment. In the NP condition, Pi concentration of all seedlings was improved, but there was still no significant difference. However, wild type and complemented knockout mutants were significantly higher in the condition of 1 mM Pi. Similarly, Shin et al. (2004) found that the differences of Pi concentrations between *pht1.1* and wild type were significant under 0.5 and 1 mM Pi treatments but not significant under 0.1 and 0.2 mM Pi treatments. The phenotype especially the root architecture of *pht1.1* and complemented *pht1.1* was not significantly different under LP treatments (Supplementary Figure 2). Also, there was no significant difference between *pht1.1* and complemented *pht1.1* under NP and HP conditions (data were not shown). Yeast mutant EY1707 is a defect strain of phosphate uptake. It can grow normally in YNB medium containing galactose but cannot grow in YNB containing glucose. *MtPT6* could contribute to the growth of yeast mutant EY1707 in YNB containing glucose (Supplementary Figure 3). All these results suggested that *MtPT6* plays a role in Pi absorbance.

## Discussion

Pi is one of the most essential macroelements, composed of 2‰ of dry weight of plant cell, and thus plants need to absorb lots of Pi to maintain their growth (Bieleski and Ferguson, 1983; Schachtman et al., 1998). PHT1 family genes are mainly expressed in roots and responsible for Pi absorbance and transport from roots to shoots (Bucher et al., 2001; Mudge et al., 2002). Symbiosis between rhizobium and legume plays a vital role not only in plant growth but also in conservation of the ecosystem. Phosphate is required for the symbiosis process, and it is preferentially translocated from other organs into nodules to maintain N_2_ fixation under low-phosphate stress (Thuynsma et al., 2014). So, phosphate transporters should be essential for symbiosis.

Only five members of the PHT1 family of *M. truncatula* were reported (Liu et al., 1998, 2008; Chiou et al., 2001; Harrison et al., 2002; Javot et al., 2007). However, we found 11 members in the MtPT1 family by blasting in NCBI. As shown by qRT-PCR results (Figure 2), most genes of the MtPT1 family were highly expressed in root tissues especially in low phosphate conditions, suggesting that these genes play an important role in phosphate uptake by roots. Some genes were also expressed in shoots and induced by low-phosphate stress such as *MtPT5*, *MtPT6*, *MtPT7*, and *MtPT8*. This suggests that the MtPT1 family also play roles in phosphate translocation (Mudge et al., 2002; Wang et al., 2014). As for nodule tissues, the expression levels of *MtPT5*, *MtPT6*, and *MtPT7* were relatively higher. *MtPT7* was not response to low-phosphate stress, suggesting it might not participate in phosphate homeostasis of the nodule. *MtPT5* and *MtPT6* were highly induced by low-phosphate stress; thus, they may play important roles in phosphate homeostasis of the nodule.

Four of nine members of the PHT1 family in *Arabidopsis* are expressed in the interface between the roots and the soil (Mudge et al., 2002; Shin et al., 2004). Histochemical GUS staining results (Figure 3) showed that *MtPT6* was expressed in root epidermis, cortical cells, and vascular tissue, as well as in root tips and nodules; subcellular localization shows that *MtPT6* protein was localized to the plasma membrane like other phosphate transporters (Nussaume et al., 2011; Remy et al., 2012; Chang et al., 2019). These reveals that it plays roles in phosphate absorbance and translocation. It was reported that both *MtPT1* and *MtPT2* were expressed in root epidermis and cortical cells, and *MtPT2* was also expressed in vascular tissue (Xiao et al., 2006). *MtPT3* was mainly expressed in vascular tissue, and *MtPT5* was expressed in epidermis, cortical cells, and root hairs (Liu et al., 2008). The overlap of expression tissue suggests that these genes are partially redundant in function. The redundant function also was shown in phylogenetic analysis because of the very close relationship among MtPT1, MtPT2, and MtPT3. Moreover, *MtPT6* was strongly induced by low-phosphate stress not only in roots but also in nodules, which is similar to the results of qRT-PCR of *MtPT6*. In soybean, two genes have been reported to function in maintaining phosphate homeostasis of nodules. *GmPT5* was mainly expressed in nodule vascular bundles, transporting Pi from the root vascular system into nodules (Qin et al., 2012). *GmPT7* could be upregulated by low phosphate treatments and was localized in the outer cortex and fixation zone; it could uptake Pi from soil and translocate to the fixation zone (Chen et al., 2019). In our study, *MtPT6* was almost expressed in the whole nodule including vascular bundles, cortex, and fixation zone. So, *MtPT6* may play functions in Pi uptake from the soil and transporting Pi from other tissues to nodules by root vascular systems. However, the specific role and mechanism of *MtPT6* in homeostasis of nodules need to be studied further.

Arsenate is extensive in soil, and AsO_4_^3–^ is structurally similar to phosphate; thus, phosphate transporters can absorb not only Pi but also arsenate (Fitz and Wenzel, 2002). AsO_4_^3–^ will be reduced to AsO_3_^3–^ immediately in plant cell, and AsO_3_^3–^ is toxic to cells (Fitz and Wenzel, 2002). *Arabidopsis* knockout mutant *pht1.1* and *pht1.4* are resistant to As treatment, although there are no significantly different phenotypes between wild type and knockout mutants under different Pi concentration conditions (Shin et al., 2004). After overexpressing *MtPT6* in these two mutants, transgenic plants became more susceptible to As treatment. There was no significant difference of phosphate content between *pht1.1* knockout mutant and *MtPT6* complemented *pht1.1* under LP and NP conditions. However, overexpressing *MtPT6* could significantly improve the Pi content of *pht1.1* under high phosphate conditions. These results suggest that under relative low phosphate conditions, *MtPT6* contributes to the acquisition of Pi but that other phosphate transporters also make a significant contribution. However, *MtPT6* appears to play a major role in phosphate acquisition under high phosphate conditions.

In conclusion, the expression patterns of *PHT1* family members were characterized successfully in various tissues of *M. truncatula* under normal and low phosphate conditions. *MtPT6* was expressed in shoots, roots, and nodules and responded to low-phosphate stress. Moreover, *MtPT6* could improve phosphate acquisition in transgenic *Arabidopsis*.

## Data Availability Statement

The original contributions presented in the study are included in the article/Supplementary Material, further inquiries can be directed to the corresponding authors.

## Author Contributions

YC performed the statistical analysis and drafted the manuscript. JL and YC designed the experiments. YC, YL, JZ, SL, and YA carried out the experiments. PY and TH provided ideas and revised the manuscript. All authors approved the submitted manuscript.

## Conflict of Interest

The authors declare that the research was conducted in the absence of any commercial or financial relationships that could be construed as a potential conflict of interest.

## Footnotes

1 https://blast.ncbi.nlm.nih.gov/

## References

[B1] Ai P. Sun S. Zhao J. Fan X. Xin W. Guo Q. (2009). Two rice phosphate transporters, OsPht1; 2 and OsPht1; 6, have different functions and kinetic properties in uptake and translocation. *Plant J.* 57 798–809. 10.1111/j.1365-313X.2008.03726.x 18980647

[B2] Bieleski R. Ferguson I. (1983). “Physiology and metabolism of phosphate and its compounds,” in *Inorganic Plant Nutrition*, eds Läuchli A. Bieleski R. L. (Berlin: Springer), 422–449. 10.1007/978-3-642-68885-0_15

[B3] Bucher M. Rausch C. Daram P. (2001). Molecular and biochemical mechanisms of phosphorus uptake into plants. *J. Plant Nutr. Soil Sci.* 164 209–217.

[B4] Chabaud M. Boisson-Dernier A. Zhang J. Taylor C. G. Yu O. Barker D. G. (2006). “Agrobacterium rhizogenes-mediated root transformation,” in *The Medicago truncatula Handbook*, eds Journer U. Mathesius E. P. Sumner L. W. (Ardmore, OK: Samuel Roberts Noble Foundation). Available online at: http://www.noble.org/MedicagoHandbook

[B5] Chang M. X. Gu M. Xia Y. W. Dai X. L. Dai C. R. Zhang J. (2019). *OsPHT1*; 3 mediates uptake, translocation, and remobilization of phosphate under extremely low phosphate regimes. *Plant Physiol.* 179 656–670. 10.1104/pp.18.01097 30567970 PMC6426419

[B6] Chen L. Qin L. Zhou L. Li X. Chen Z. Sun L. (2019). A nodule-localized phosphate transporter GmPT7 plays an important role in enhancing symbiotic N2 fixation and yield in soybean. *New Phytol.* 221 2013–2025. 10.1111/nph.15541 30317659

[B7] Chiou T. J. Liu H. Harrison M. J. (2001). The spatial expression patterns of a phosphate transporter (MtPT1) from *Medicago truncatula* indicate a role in phosphate transport at the root/soil interface. *Plant J.* 25 281–293. 10.1046/j.1365-313x.2001.00963.x 11208020

[B8] Davis A. M. Hall A. Millar A. J. Darrah C. Davis S. J. (2009). Protocol: streamlined subprotocols for floral-dip transformation and selection of transformants in *Arabidopsis thaliana*. *Plant Methods* 5:3. 10.1186/1746-4811-5-3 19250520 PMC2660325

[B9] Domagalski J. Lin C. Luo Y. Kang J. Wang S. Brown L. R. (2007). Eutrophication study at the Panjiakou-Daheiting reservoir system, northern Hebei province, People’s Republic of China : chlorophyll-a model and sources of phosphorus and nitrogen. *Agric. Water. Manag.* 94 43–53. 10.1016/j.agwat.2007.08.002

[B10] Fitz W. J. Wenzel W. W. (2002). Arsenic transformations in the soil-rhizosphere-plant system: fundamentals and potential application to phytoremediation. *J. Biotechnol.* 99 259–278. 10.1016/S0168-1656(02)00218-3 12385714

[B11] Harrison M. J. Dewbre G. R. Liu J. (2002). A phosphate transporter from *Medicago truncatula* involved in the acquisition of phosphate released by arbuscular mycorrhizal fungi. *Plant Cell.* 14 2413–2429. 10.1105/tpc.004861 12368495 PMC151226

[B12] Hinsinger P. (2001). Bioavailability of soil inorganic P in the rhizosphere as affected by root-induced chemical changes: a review. *Plant Soil* 237 173–195. 10.1023/A:1013351617532

[B13] Horst M. (2012). *Mineral Nutrition of Higher Plants*, 2nd Edn, Cambridge, MA: Academic Press.

[B14] Javot H. Penmetsa R. V. Terzaghi N. Cook D. R. Harrison M. J. (2007). A *Medicago truncatula* phosphate transporter indispensable for the arbuscular mycorrhizal symbiosis. *Proc. Natl. Acad. Sci. U.S.A.* 104 1720–1725. 10.1073/pnas.0608136104 17242358 PMC1785290

[B15] Jia H. Ren H. Gu M. Zhao J. Sun S. Zhang X. (2011). The phosphate transporter gene OsPht1; 8 is involved in phosphate homeostasis in rice. *Plant Physiol.* 156 1164–1175. 10.1104/pp.111.175240 21502185 PMC3135946

[B16] John M. K. (1970). Colorimetric determination of phosphorus in soil and plant materials with ascorbic acid. *Soil Sci.* 109 214–220.

[B17] Jones D. T. Taylor W. R. Thornton J. M. (1992). The rapid generation of mutation data matrices from protein sequences. *Bioinformatics* 8 275–282. 10.1093/bioinformatics/8.3.275 1633570

[B18] Karthikeyan A. S. Varadarajan D. K. Mukatira U. T. Matilde Paino D. U. Barbara D. Raghothama K. G. (2002). Regulated expression of *Arabidopsis* phosphate transporters. *Plant Physiol.* 130:221. 10.1104/pp.020007 12226502 PMC166555

[B19] Liu F. Chang X. Ye Y. Xie W. Wu P. Lian X. (2011). Comprehensive sequence and whole-life-cycle expression profile analysis of the phosphate transporter gene family in rice. *Mol. Plant* 4 1105–1122. 10.1093/mp/ssr058 21832284

[B20] Liu H. Trieu A. T. Blaylock L. A. Harrison M. J. (1998). Cloning and characterization of two phosphate transporters from *Medicago truncatula* roots: regulation in response to phosphate and to colonization by Arbuscular mycorrhizal (AM) Fungi. *Mol. Plant Microb. Interact.* 11 14–22. 10.1094/MPMI.1998.11.1.14 9425684

[B21] Liu J. Versaw W. K. Pumplin N. Gomez S. K. Blaylock L. A. Harrison M. J. (2008). Closely related members of the *Medicago truncatula* PHT1 phosphate transporter gene family encode phosphate transporters with distinct biochemical activities. *J. Biol. Chem.* 283:2008. 10.1074/jbc.M802695200 18596039 PMC3259825

[B22] Liu J. Yang L. Luan M. Wang Y. Zhang C. Zhang B. (2015). A vacuolar phosphate transporter essential for phosphate homeostasis in *Arabidopsis*. *Proc. Natl. Acad. Sci. U.S.A.* 112 E6571–E6578. 10.1073/pnas.1514598112 26554016 PMC4664319

[B23] Lizbeth L. D. Antonio L. M. Isabel G. S. José L. Luis H. (2014). Phosphate nutrition: improving low-phosphate tolerance in crops. *Annu. Rev. Plant Biol.* 65 95–123. 10.1146/annurev-arplant-050213-035949 24579991

[B24] Lu M. Cheng Z. Zhang X.-M. Huang P. Fan C. Yu G. (2020). Spatial divergence of PHR-PHT1 modules maintains phosphorus homeostasis in soybean nodules. *Plant Physiol.* 184 236–250. 10.1104/pp.19.01209 32680974 PMC7479890

[B25] Mudge S. R. Rae A. L. Diatloff E. Smith F. W. (2002). Expression analysis suggests novel roles for members of the Pht1 family of phosphate transporters in *Arabidopsis*. *Plant J.* 31 341–353. 10.1046/j.1365-313X.2002.01356.x 12164813

[B26] Nagy R. Vasconcelos M. J. V. Zhao S. McElver J. Bruce W. Amrhein N. (2006). Differential regulation of five Pht1 phosphate transporters from maize (*Zea mays* L.). *Plant Biol.* 8 186–197. 10.1055/s-2005-873052 16547863

[B27] Nussaume L. Kanno S. Javot H. Marin E. Nakanishi T. M. Thibaud M. C. (2011). Phosphate import in plants: focus on the PHT1 transporters. *Front. Plant Sci.* 2:83. 10.3389/fpls.2011.00083 22645553 PMC3355772

[B28] Paszkowski U. Kroken S. Roux C. Briggs S. P. (2002). Rice phosphate transporters include an evolutionarily divergent gene specifically activated in arbuscular mycorrhizal symbiosis. *Proc. Natl. Acad. Sci. U.S.A.* 99 13324–13329. 10.1073/pnas.202474599 12271140 PMC130632

[B29] Preuss C. P. Huang C. Y. Gilliham M. Tyerman S. D. (2010). Channel-like characteristics of the low-affinity barley phosphate transporter PHT1; 6 when expressed in *Xenopus oocytes*. *Plant Physiol.* 152 1431–1441. 10.1104/pp.109.152009 20053709 PMC2832247

[B30] Qin L. Zhao J. Tian J. Chen L. Sun Z. Guo Y. (2012). The high-affinity phosphate transporter GmPT5 regulates phosphate transport to nodules and nodulation in soybean. *Plant Physiol.* 159 1634–1643. 10.1104/pp.112.199786 22740613 PMC3425202

[B31] Remy E. Cabrito T. Batista R. Teixeira M. Sá-Correia I. Duque P. (2012). The Pht1;9 and Pht1;8 transporters mediate inorganic phosphate acquisition by the *Arabidopsis thaliana* root during phosphorus starvation. *New Phytol.* 195 356–371. 10.1111/j.1469-8137.2012.04167.x 22578268

[B32] Ren F. Zhao C. Z. Liu C. S. Huang K. L. Guo Q. Q. Chang L. L. (2014). A *Brassica napus* PHT1 phosphate transporter, BnPht1; 4, promotes phosphate uptake and affects roots architecture of transgenic *Arabidopsis*. *Plant Mol. Biol.* 86 595–607. 10.1007/s11103-014-0249-y 25194430

[B33] Schachtman D. P. Reid R. J. Ayling S. M. (1998). Phosphorus uptake by plants: from soil to cell. *Plant Physiol.* 116 447–453. 10.1104/pp.116.2.447 9490752 PMC1539172

[B34] Shin H. Shin H. S. Dewbre G. R. Harrison M. J. (2004). Phosphate transport in *Arabidopsis*: Pht1;1 and Pht1;4 play a major role in phosphate acquisition from both low- and high- phosphate environments. *Plant J.* 39 629–642. 10.1111/j.1365-313X.2004.02161.x 15272879

[B35] Stecher G. Tamura K. Kumar S. (2020). Molecular evolutionary genetics analysis (MEGA) for macOS. *Mol. Biol. Evol.* 37 1237–1239. 10.1093/molbev/msz312 31904846 PMC7086165

[B36] Sun S. Gu M. Cao Y. Huang X. Zhang X. Ai P. (2012). A constitutive expressed phosphate transporter, OsPht1; 1, modulates phosphate uptake and translocation in phosphate-replete rice. *Plant Physiol.* 159 1571–1581. 10.1104/pp.112.196345 22649273 PMC3425197

[B37] Teng W. Zhao Y. Y. Zhao X. Q. He X. Ma W. Y. Deng Y. (2017). Genome-wide identification, characterization, and expression analysis of PHT1 phosphate transporters in wheat. *Front. Plant Sci.* 8:543. 10.3389/fpls.2017.00543 28443126 PMC5386973

[B38] Thuynsma R. Valentine A. Kleinert A. (2014). Phosphorus deficiency affects the allocation of below-ground resources to combined cluster roots and nodules in *Lupinus albus*. *J. Plant Physiol.* 171 285–291. 10.1016/j.jplph.2013.09.001 24129121

[B39] Tocquin P. Corbesier L. Havelange A. Pieltain A. Kurtem E. Bernier G. (2003). A novel high efficiency, low maintenance, hydroponic system for synchronous growth and flowering of *Arabidopsis thaliana*. *BMC Plant Biol.* 3:2. 10.1186/1471-2229-3-2 12556248 PMC150571

[B40] Vance C. P. (2010). Quantitative trait loci, epigenetics, sugars, and microRNAs: quaternaries in phosphate acquisition and use. *Plant Physiol.* 154 582–588. 10.1104/pp.110.161067 20921189 PMC2949005

[B41] Wang X. Wang Y. Piñeros M. A. Wang Z. Wang W. Li C. (2014). Phosphate transporters OsPHT1;9 and OsPHT1;10 are involved in phosphate uptake in rice. *Plant Cell Environ.* 37 1159–1170. 10.1111/pce.12224 24344809

[B42] Xiao K. Liu J. Dewbre G. Harrison M. Wang Z.-Y. (2006). Isolation and characterization of root-specific phosphate transporter promoters from *Medicago truncatula*. *Plant Biol.* 8 439–449. 10.1055/s-2005-873053 16917979

