# Supplementary figures and images for: Functional Analysis of the Phosphate Transporter Gene MtPT6 From Medicago truncatula
Source: Front Plant Sci. 2021 Feb 4;11:620377. doi: 10.3389/fpls.2020.620377 (PMC7890022; doi:10.3389/fpls.2020.620377)

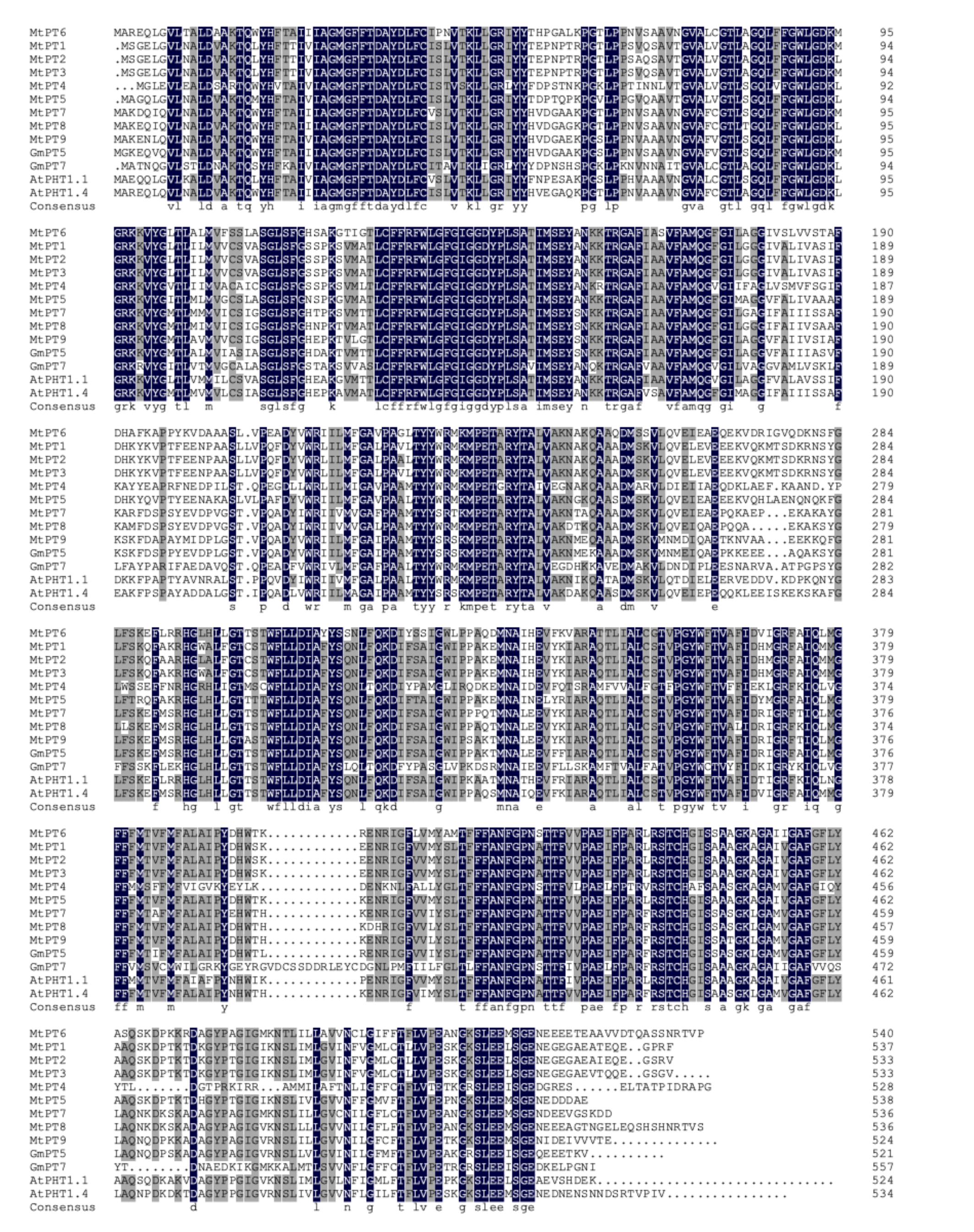

Supplement: Supplementary file 1 [file Image_1.TIFF]

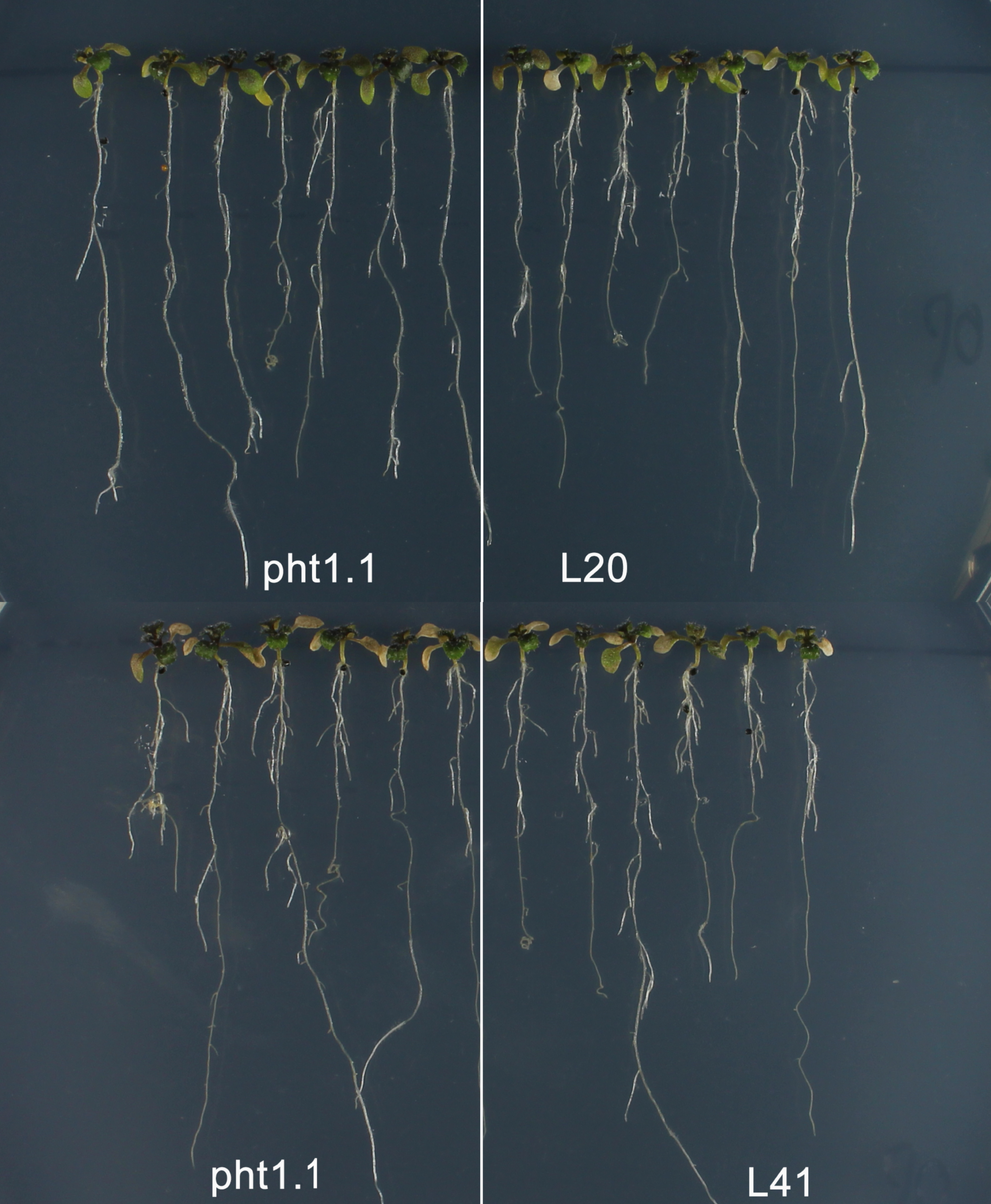

Supplement: Supplementary file 2 [file Image_2.TIFF]

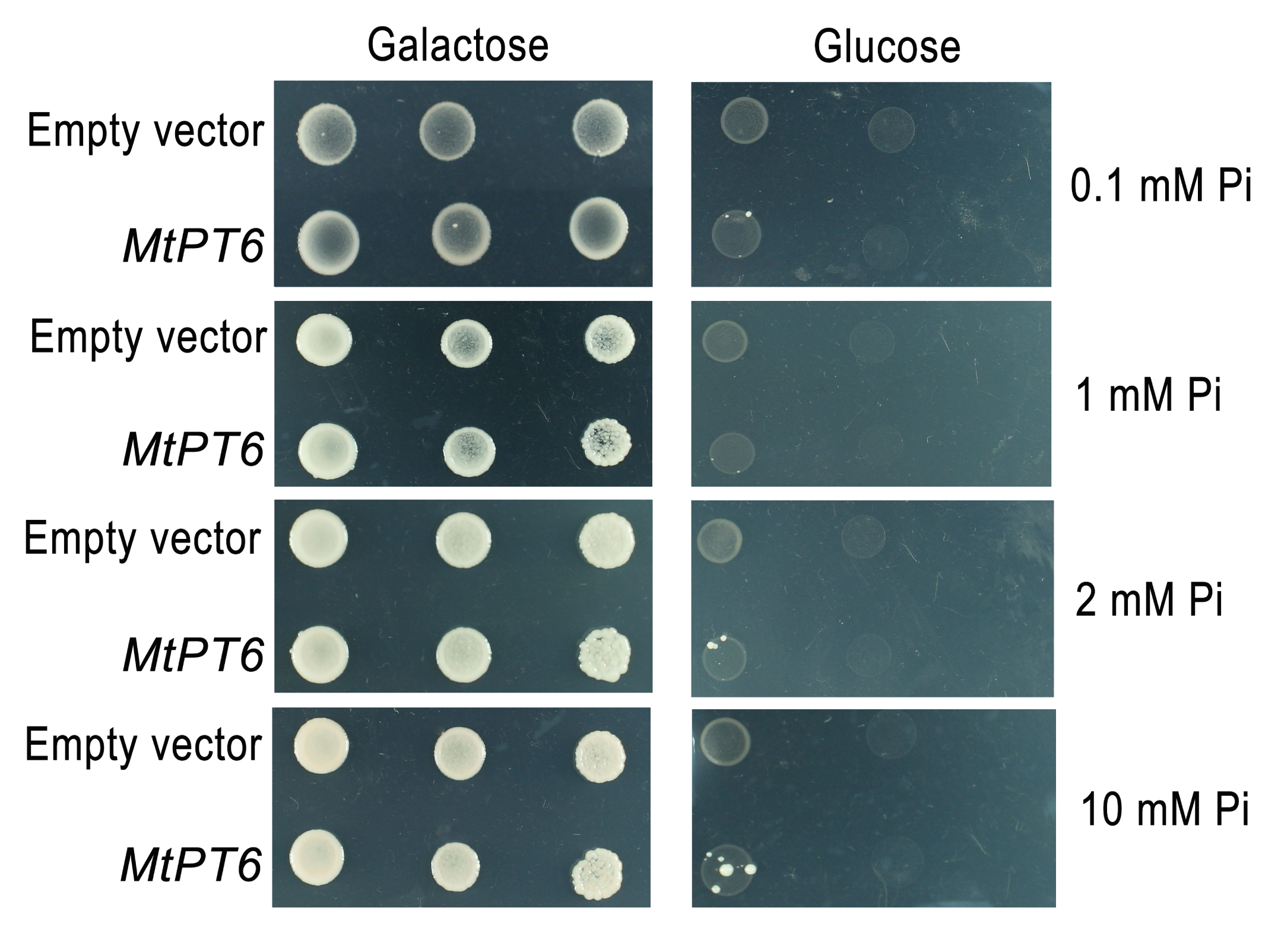

Supplement: Supplementary file 3 [file Image_3.TIFF]
