# Supplementary material for: Functional Analysis of the Phosphate Transporter Gene MtPT6 From Medicago truncatula
Source: Front Plant Sci. 2021 Feb 4;11:620377. doi: 10.3389/fpls.2020.620377 (PMC7890022; doi:10.3389/fpls.2020.620377)
Supplement: Supplementary file 4 [file Table_1.DOCX]

Supplementary Material

**Supplementary Figure 1.** Amino acid sequence alignment of PHT1 family genes from *M. truncatula* with *Arabisopsis* and soybean. Conserved residues are shaded in dark blue color; similar residues in more than 75 % are shaded in grey color.

**Supplementary Figure 2.** Effects of *MtPT6* on seedlings development of *pht1.1* in low phosphate condition. *Arabidopsis* knockout mutant *pht1.1* and complemented *pht1.1* with *MtPT6* (L20 and L41) lines were grown in 1/2 MS medium plates containing normal Pi (625 µM KH_2_PO_4_) for one week and then were transformed to low Pi conditions (5 µM KH_2_PO_4_) for one week.

**Supplementary Figure 3.** Functional characterization of *MtPT6* in yeast mutant EY1707. EY1707, highly defective in phosphate uptake, can grow normally in YNB medium containing galactose but not grow in YNB containing glucose. pRS426 vector is used to express *MtPT6* in yeast. 5 μl of 10-fold serial dilution with the original cell number of 6 x 10^5^ were applied to SG-Trp/Ura and SD-Trp/Ura medium supplied with 0.1-10 mM Pi and then incubated at 30 °C for 4 d.
